# The role of non-structural carbohydrates in tree physiology and forest management: a comprehensive review

**DOI:** 10.3389/fpls.2026.1790037

**Published:** 2026-06-11

**Authors:** Gaoyan Huang, Yue Rong, Yan Huang, Zhuizhui Guan, Tianxiao Ma, Yanmei Wang, Nowsherwan Zarif

**Affiliations:** 1 Henan Agricultural University, Zhengzhou, China; 2 Economic Crop Promotion Station of Henan Province, Zhengzhou, China; 3 Xinyang Agriculture and Forestry University, Xinyang, China; 4 Pakistan Forest Institute, Peshawar, Pakistan

**Keywords:** arbor NSC, carbon sequestration, climate change, forest management, non-structural carbohydrates (NSCs), remote sensing, spatial-temporal dynamics, tree physiology

## Abstract

Non-structural carbohydrates (NSCs) in trees, comprising soluble sugars, starch, and other labile compounds, are integral to tree physiology, resilience against environmental stressors, and the dynamics of ecosystem carbon. This review consolidates existing knowledge on the definition, forms, and functions of NSCs, examines their historical significance in forest management, and evaluates the impact of contemporary forestry practices on NSC storage. It understands the spatial and temporal dynamics of NSCs, including variability among forest types, topographic influences, and seasonal patterns, and underscores the use of remote sensing and GIS technologies in mapping carbon distribution. Additionally, the review assesses long-term trends in carbon allocation influenced by climate change and phenological variations, and proposes future strategies for incorporating carbon management into sustainable forestry. It also identifies key knowledge gaps, such as uncertainties in NSC turnover rates and the interactive effects of multiple stressors, to inform future research endeavors. This review underscores the significance of NSCs in sustaining forest health and augmenting carbon sequestration within the context of a changing global environment by integrating physiological insights with practical management strategies.

## Graphical Abstract

**Figure:**
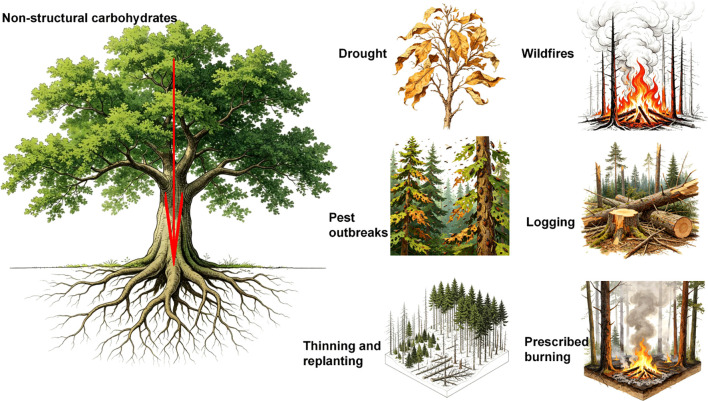

## 1 Introduction

Non-structural carbohydrates (NSCs)—primarily soluble sugars and starch—serve as the central carbon currency mediating tree growth, survival, and resilience to environmental stress (Zhou et al., 2025; Tu et al., 2025; Wang et al., 2025). Despite decades of research, a unifying framework that integrates the production, mobilization, and storage of NSCs across scales—from cellular physiology to forest management—remains lacking. Here we propose a source–sink–storage dynamic balance model, which conceptualizes NSCs as a buffered pool balancing carbon supply (source), demand (sink), and reserve (storage). This framework provides a dual perspective: NSCs function not only as substrates for immediate metabolic needs but also as a carbon buffer that sustains tree function during stress-induced supply–demand mismatches (Fu et al., 2025; Guo et al., 2025). We organize this review around four interconnected themes. First, we examine the physiological roles of NSCs, focusing on source–sink relationships and how allocation trade-offs influence tree performance. Second, we address measurement challenges and the spatiotemporal dynamics of NSCs, emphasizing their storage characteristics across organs, seasons, and forest types. Third, we evaluate how contemporary forest management practices—including selective logging, thinning, prescribed burning, and afforestation—alter NSC storage, leading to its dysregulation or recovery under multiple stressors. Finally, we discuss predictive modeling efforts and future research directions needed to integrate NSC dynamics into sustainable forest management under global environmental change. By anchoring this review in the source–sink–storage balance framework, we aim to bridge physiological insights with practical management strategies and highlight critical knowledge gaps, including uncertainties in NSC turnover rates, interactive effects of co-occurring stressors, and challenges in scaling from individual trees to ecosystems.

## 2 The NSC in arbor trees

### 2.1 Collection and screening of literature

This review follows the PRISMA (Preferred Reporting Items for Systematic Reviews and Meta-Analyses) framework to ensure transparency and reproducibility. A systematic literature search was conducted in Web of Science, Scopus, and Google Scholar for peer-reviewed articles published between January 2000 and December 2025, using keywords such as ‘non-structural carbohydrates,’ ‘tree physiology,’ ‘forest management,’ ‘carbon dynamics,’ and ‘climate change.’ Studies were included if they focused on tree NSC dynamics, carbon allocation, or management impacts in forest ecosystems, and excluded if they lacked empirical data, were purely modeling without validation, or were non-English or grey literature. The initial search yielded 1247 records, of which 278 duplicates were removed. After title and abstract screening, 402 studies were excluded based on irrelevance, leaving 567 full-text articles assessed for eligibility. Following full-text screening against inclusion/exclusion criteria, a total of 83 studies were finally included in the qualitative synthesis of this review.

### 2.2 Definition, forms, and dynamics of NSC in trees

In arboreal systems, NSCs denote labile carbon compounds that are not incorporated into structural biomass, such as cell walls, but instead serve as energy reserves or substrates for metabolic processes, participating in various life activities. The primary forms of NSCs include soluble sugars—namely glucose, fructose, and sucrose—and starch, with minor contributions from sugar alcohols and lipids in certain species (Richardson et al., 2015; Hartmann and Trumbore, 2016; Landhäusser et al., 2018; Schoonmaker et al., 2021). A two-pool model of NSC dynamics, which differentiates between fast-cycling pools (soluble sugars with a turnover of less than one year) and slow-cycling pools (starch in older tissues with a turnover exceeding five years), has been validated through the use of radiocarbon (¹^+^C) bomb spike data (Richardson et al., 2015). For example, in temperate trees, NSCs located in branches and the outer growth rings of stemwood exhibit a ¹^+^C signature that aligns with the current growing season. In contrast, NSCs in older aboveground and belowground tissues are enriched in ¹^+^C, suggesting the utilization of older assimilates (Richardson et al., 2015). In *Acer saccharum*, the sap mobilized during early spring originates from a well-mixed NSC pool, exhibiting a mean turnover time of 3–5 years, as determined by radiocarbon (¹^+^C) analysis (Muhr et al., 2016). There is significant spatial variability in NSC concentrations within tree crowns. For instance, in *Juglans mandshurica* and *Ulmus japonica*, older branches with smaller basal diameters have NSC concentrations that are 2–3 times higher than those in larger branches, whereas leaves and new twigs exhibit minimal variation with respect to branch characteristics (Cheng and Wang, 2015). This variability underscores the necessity for targeted sampling protocols, such as employing 3-cm-diameter branches to accurately scale crown NSC content, thereby potentially reducing estimation errors by up to 40% (Cheng and Wang, 2015).

The relationship between NSC dynamics and tree survival under stress conditions is intricate. In Amazonian trees subjected to complete stem girdling, survival over several months is sustained by stored carbon that is over a decade old, as evidenced by radiocarbon (¹^+^C) analysis indicating the utilization of NSC fixed up to 13–14 years prior (Muhr et al., 2018). Similarly, European beech (*Fagus sylvatica*) trees, when defoliated by late frost, mobilize NSC with an average age of five years to sustain metabolic functions during leafless periods, during which soluble sugar concentrations decline by 30% before the canopy recovers (D'Andrea et al., 2019). The “last-in, first-out” pattern of NSC mobilization—where recently fixed carbon is utilized first, followed by older reserves—has been modeled using probability distributions of NSC age and transit times (Herrera-Ramírez et al., 2020). This modeling demonstrates that species with differing leaf phenology, such as *Pinus halepensis* and *Acer rubrum*, exhibit distinct NSC turnover dynamics (Herrera-Ramírez et al., 2020). For instance, *Pinus taeda* (loblolly pine) exhibits an average NSC transit time of 2.5 years, in contrast to 4.2 years observed in *P. halepensis*, which indicates variations in root storage allocation (Herrera-Ramírez et al., 2020). During drought conditions, NSC partitioning undergoes a shift: starch concentrations across all organs decrease by 20–50% among various tree species, whereas soluble sugar levels in leaves increase by 15–30%, particularly under severe drought stress (He et al., 2020). In the case of apple trees (*Malus domestica*), a substantial crop load (sink demand) results in a 25% reduction in leaf NSC concentrations and downregulates photosynthetic capacity, thereby demonstrating the feedback mechanism between NSC status and source activity (Ngao et al., 2021).

### 2.3 Physiological and ecological functions of NSC in trees

NSCs fulfill three primary physiological roles: energy storage for growth and stress response, osmoregulation, and phloem transport. In boreal conifers, seasonal transitions from starch to sugars, exemplified by a 60% increase in stemwood sugar concentrations in *Picea glauca* during winter, enhance cold tolerance by reducing the freezing point of xylem sap (Schoonmaker et al., 2021). In the case of *Pinus contorta* (lodgepole pine) affected by mountain pine beetle infestations, NSC concentrations initially decrease in the attacked bole region (by 35% within six months), followed by subsequent reductions in the needles (a 20% decline by late 2011) and roots (a 15% decline by 2012) (Wiley et al., 2016). Trees with higher initial bark sugar concentrations (≥8% dry weight) exhibited 50% less beetle damage, indicating that NSC-mediated defense mechanisms, such as osmotic adjustment or the production of secondary metabolites, may play a role (Wiley et al., 2016). In *Pinus flexilis* seedlings experiencing drought stress, the depletion of non-structural carbohydrates (NSC)—evidenced by a 30% reduction in foliar NSC—has been associated with hydraulic failure. This process is characterized by an initial 40% decline in starch reserves, followed by the utilization of soluble sugars (Reinhardt et al., 2015). Conversely, in *F. sylvatica* saplings, the allocation of NSC to storage is maintained as a priority even under conditions of severe carbon limitation, such as reduced CO_2_ levels combined with drought. This is demonstrated by ¹³C labeling, which indicates a sustained carbon flow to storage pools despite a negative net carbon gain (Hartmann et al., 2015).

From an ecological standpoint, the dynamics of non-structural carbohydrates (NSCs) play a crucial role in mediating species interactions and influencing ecosystem resilience. In tropical rainforests, the practice of selective logging leads to a reduction in foliar NSC concentrations by 15–20%, primarily due to decreased nutrient availability. This alteration in nutrient dynamics facilitates a shift in community composition toward pioneer species, which are characterized by elevated foliar phosphorus concentrations (Swinfield et al., 2020). Furthermore, tree species diversity contributes to the stabilization of soil carbon by reducing the temperature sensitivity (Q_10_) of soil respiration. Specifically, Q_10_ values are observed to be 20–30% lower in stands with high species diversity compared to monocultures (Luan et al., 2018). This phenomenon is linked to NSC-mediated modifications in microbial community composition; high-diversity stands demonstrate a 15% increase in the fungal-to-bacterial ratio, which in turn decreases the decomposition rates of recalcitrant organic matter (Luan et al., 2018). In Mediterranean oak (*Quercus frainetto*) forests, trees exhibiting decline demonstrate a 9.7% reduction in intrinsic water-use efficiency and elevated δ¹^8^O values, suggesting increased water loss and depletion of non-structural carbohydrates (NSC) compared to their non-declining counterparts (Colangelo et al., 2017). NSC also plays a critical role in plant-soil feedback mechanisms: in afforested regions on the Qinghai Plateau, the accumulation of NSC in tree roots enhances colonization by arbuscular mycorrhizal fungi (AMF) by 30%, which in turn facilitates a 12% increase in soil organic carbon (SOC) sequestration (Shi et al., 2015).

### 2.4 Measurement techniques and challenges in quantifying NSC

Traditional extraction-based techniques for quantifying NSC, such as solvent extraction (e.g., ethanol for soluble sugars) and enzymatic hydrolysis (e.g., amyloglucosidase for starch), encounter challenges related to low specificity and the potential for overestimation. In the case of trembling aspen (*Populus tremuloides*), these methods have been shown to overestimate sugar carbon by 273 ± 101%, with overestimations reaching as high as 480% in heartwood. This discrepancy arises because these methods also account for non-sugar soluble carbon and metabolically inactive carbon from dead tissues (Peltier et al., 2023). A more precise alternative is the incubation method, which measures the ¹^+^C of respired CO_2_ from living tissues, thereby providing a more accurate estimation of metabolically available NSC. This method demonstrates that NSC recovery varies significantly, ranging from 3% in heartwood to 85% in shallow sapwood, with ¹^+^C signatures corresponding with extraction-based results in sapwood but exhibiting substantial divergence in heartwood (Δ¹^+^C differences of 15–20‰) (Peltier et al., 2023). The measurement of NSC is further complicated by diurnal fluctuations; for instance, in almond (*Prunus dulcis*) trees, leaf starch reserves are completely depleted overnight, while starch levels in woody tissues fluctuate by more than 50% over a 24-hour period (Tixier et al., 2018). To mitigate this variability, implementing standardized sampling protocols—such as collecting samples at midday during the growing season—can decrease measurement variability by 30–40% (Tixier et al., 2018).

Radiocarbon analysis is a highly effective technique for estimating the turnover times of NSC. In two temperate tree species, the average age of NSC in the outer stemwood growth rings is less than one year, whereas in older rings, it exceeds five years (Richardson et al., 2015). In *Pinus edulis*, drought-induced mortality is associated with a 30% reduction in foliar NSC, with starch concentrations initially decreasing by 40% before the mobilization of soluble sugars occurs (Adams et al., 2013). Probabilistic models of NSC age distributions suggest that simulated starvation results in a flattening of the age distribution and an increase in mean transit time by 20–30%, facilitating the estimation of reserve depletion periods (Herrera-Ramírez et al., 2020). For example, *P. halepensis* exhibits an average NSC transit time of 2.5 years, indicating that it would deplete its reserves in approximately six months under conditions of complete carbon limitation (Herrera-Ramírez et al., 2020). Inter-laboratory comparisons demonstrate substantial variability in NSC measurements: in *Eucalyptus globulus* leaves, soluble sugar concentrations range from 23 to 116 mg g^-^¹ across 29 laboratories, while starch estimates vary by an order of magnitude (6–533 mg g^-^¹) (Quentin et al., 2015). This variability underscores the necessity of implementing standardized reference methods, such as the phenol-sulfuric acid assay for sugars, which can reduce inter-laboratory discrepancies by up to 70% (Quentin et al., 2015).

## 3 Historical perspectives on forest management and NSC

### 3.1 Historical evolution and climate-driven adaptation of forest management practices influencing NSC dynamics

Forest management practices have transitioned from extractive logging methods to ecosystem-based approaches, significantly affecting NSC dynamics. Prior to the 20th century, selective logging in European forests primarily targeted the removal of large-diameter trees, which led to a reduction in stand basal area by 30–40% and a depletion of NSC reserves in the remaining trees by 20% (Fuchs et al., 2016). During the mid-20th century, the implementation of clear-cutting practices eradicated NSC storage in aboveground biomass and disrupted belowground NSC cycling, resulting in a 50% decline in soil microbial biomass (Thom and Seidl, 2016). By the 1980s, the concept of sustainable forest management (SFM) had gained prominence, focusing on the retention of residual trees and deadwood. For example, in Finnish boreal forests, the retention of 10–15% of stand volume resulted in a 15–20% increase in non-structural carbohydrate (NSC) storage in residual trees and enhanced soil NSC input via leaf litter by 30% (Akujärvi et al., 2016). In the 21st century, carbon management has been incorporated into forestry practices. Specifically, REDD+ (Reducing Emissions from Deforestation and Forest Degradation) initiatives in the Democratic Republic of Congo (DRC) have successfully reduced deforestation rates by 15% and augmented NSC storage in remaining forests by 10% (Galford et al., 2015). In Gabon, the implementation of reduced-impact logging (RIL) techniques—such as directional felling and the planning of skid trails—has preserved 85% of pre-logging NSC storage in live trees, in contrast to only 60% retention under conventional logging methods (Ndjondo et al., 2014) (**Table 1**).

**Table 1:** Key research directions related to tree NSC studies.

Author	Tree species or forests	Year	Key topic
Ndjondo et al.	Central Africa forests	2014	Effects of thinning on forests NSC
Xu et al.	*Cunninghamia lanceolata*	2014	
Galford et al.	Tropical forests	2015	
Fuchs et al.	Europe forests	2016	
Swinfield et al.	Tropical forests	2020	
Murdiyarso et al.	Mangrove forests	2021	
Xu et al.	*Cunninghamia lanceolata*	2014	Effects of fertilization on forests NSC
Shi et al.	Qinghai Plateau forests	2015	Effects of afforestation on forests NSC
Fang et al.	Forests of subtropical China	2015	
Cong et al.	Forests of Northeast China Plain	2015	
Kim et al.	*Liquidambar styraciflua*	2015	Effects of climate change on forests NSC
Liu et al.	Canada’s boreal forests	2023	
Vacek et al.	European forests	2023	
Asner and Martin	Tropical forests	2016	Spatial variability of forests NSC
Liu et al.	Broad-leaved evergreen forest of Yunnan Province	2018	
de la Cruz-Amo et al.	Andean tropical montane forests	2020	
Schoonmaker et al.	*Pinus contorta* and Picea glauca	2021	Temporal variability of forests NSC
Smith et al.	*Eucalyptus obliqua*	2018	
Levick et al.	Temperate deciduous forest	2016	GIS and NSC
Schillaci et al.	Sicily forests	2017	
Hoyos-Santillan et al.	Mangrove forests of Parita and Panama.	2025	

Climate change has significantly influenced forest management practices. In Swiss mountain forests, thinning to reduce stand density enhances NSC storage in the remaining trees by 10–15% and improves drought resilience, as evidenced by a 20% increase in pre-dawn leaf water potential (Thrippleton et al., 2023). In the Pacific Northwest, prescribed burning to decrease fuel loads elevates NSC concentrations in surviving trees by 5–10% due to diminished resource competition (Carlson et al., 2017). Nonetheless, certain practices yield unintended outcomes: in Chinese fir (*Cunninghamia lanceolata*) plantations, intensive thinning (50% intensity) results in a 17% reduction in NSC storage in the short term, although recovery to pre-thinning levels is observed within eight years (Xu et al., 2014). The evolution of management practices underscores an increasing awareness of the critical role of NSC in tree survival and carbon sequestration. Contemporary strategies emphasize the preservation of NSC reserves by minimizing disturbance intensity and retaining legacy trees, which contribute 30–40% of stand-level NSC storage (Castro-Izaguirre et al., 2016).

### 3.2 Case studies in forest management: regional variations in NSC dynamics

The transformation of Wisconsin’s forests from the 1850s to the 1930s serves as a quintessential illustration of the impact of forest management on carbon distribution. Prior to settlement, the aboveground live forest carbon (AGC) was estimated at 434 TgC; however, by the 1930s, this figure had diminished to 120 TgC as a result of agricultural clearing (Rhemtulla et al., 2009). Subsequent reforestation initiatives post-1930s facilitated a recovery of AGC to 276 TgC by the 2000s, albeit with altered spatial distribution: fire suppression has led southern savannas to store 20% more AGC than in pre-settlement times, whereas northern forests continue to exhibit a 30% deficit relative to historical levels (Rhemtulla et al., 2009). In contrast, managed beech (*F. sylvatica*) forests in Hesse, Germany, demonstrate a 16% increase in cumulative biomass production over a 19–24 year period compared to unmanaged reserves. Growth-dominance analysis reveals a more equitable biomass increment across varying tree sizes in managed stands (Krug, 2019). This phenomenon is attributed to improvements in resource-use efficiency mediated by non-structural carbohydrates (NSC); managed stands display a 15% elevation in foliar NSC concentrations, which facilitates more rapid canopy recovery following thinning (Krug, 2019).

Global case studies demonstrate considerable regional disparities in the impact of forest management on carbon dynamics. In the Brazilian Amazon, selective logging results in a 15–20% reduction in aboveground live forest carbon (AGC), while simultaneously increasing non-structural carbohydrate (NSC) storage in the remaining trees by 5–10% due to decreased competition (Piponiot et al., 2016). Conversely, in Bornean mangroves, selective logging retains 70–75% of total ecosystem carbon stocks, with soil carbon sequestration rates remaining stable at 1.2–1.5 Mg C ha^-^¹ yr^-^¹ (Murdiyarso et al., 2021). In China’s Loess Plateau, afforestation with *Robinia pseudoacacia* enhances NSC storage in tree biomass by 40–50%, augments soil NSC input by 30%, and leads to a 20% increase in soil organic carbon (SOC) sequestration (Cong et al., 2015). These instances underscore that management practices prioritizing the retention of residual trees and minimizing soil disturbance are effective in maintaining NSC dynamics and supporting long-term carbon storage.

### 3.3 Long-term temporal dynamics of NSC in response to climate change and disturbances

Long-term trends in tree carbon dynamics are influenced by the interplay of management practices, climate change, and disturbances. In the boreal forests of Canada, drought-induced tree mortality has risen by 43% since 1970, with annual biomass carbon losses averaging 1.51 ± 0.29 MgC ha^-^¹ yr^-^¹ (Liu et al., 2023). This increase in mortality is associated with the depletion of non-structural carbohydrates (NSC), as evidenced by dying trees exhibiting 30–40% lower foliar NSC concentrations compared to surviving counterparts (Liu et al., 2023). In European forests, climate change has led to a 5–10% increase in tree growth rates in northern regions, while growth has declined by 12–49% in southern regions; these variations are mediated by NSC dynamics (Vacek et al., 2023). For example, *Pinus sylvestris* in northern Europe demonstrates a 10% increase in NSC storage within stems, facilitating enhanced growth under elevated temperatures (Vacek et al., 2023).

Climate-induced phenological changes are impacting forest carbon dynamics significantly. In temperate forests, the advancement of spring leaf-out by 3–5 days per decade enhances annual carbon assimilation by 5–10%, although this benefit is somewhat mitigated by the earlier onset of autumn leaf senescence (Gu et al., 2022). In red maple (*Acer rubrum*), earlier leaf-out is correlated with increased photosynthetic carbon assimilation during the preceding growing season, with ¹³C labeling revealing a 20% higher allocation of carbon to storage pools (Gu et al., 2022). In the Swiss Alps, larch budmoth (*Zeiraphera griseana*) outbreaks affect tree-ring δ²H signatures, indicating increased mobilization of stored NSC, with δ²H enrichment ranging from 5–10‰ (Vitali et al., 2023). Over the past 700 years, these outbreaks have occurred every 8–10 years, each time decreasing aboveground live forest carbon (AGC) by 5–10% (Vitali et al., 2023).

## 4 NSC in forest ecosystems: spatio-temporal variability, influencing factors, and mapping techniques

### 4.1 Spatial and temporal variation of forest NSC

In tropical montane forests, NSC concentrations exhibit an increase with elevation, rising by 10–15% per 1000 meters, as cooler temperatures reduce respiratory losses and promote NSC storage (de la Cruz-Amo et al., 2020). In subtropical monsoon broad-leaved evergreen forests, NSC concentrations are highest in the sub-canopy (13.3% dry weight) and lowest in the understory (10.7% dry weight), with the soluble sugar-to-starch ratio peaking in the understory (0.76) (Liu et al., 2018b). This vertical variability is correlated with light availability, as sub-canopy trees exhibit photosynthetic rates that are 15% higher, thereby supporting increased NSC production (Liu et al., 2018b). Forest type exerts a significant influence on the allocation patterns of NSC. In Mediterranean evergreen forests, *Quercus ilex* allocates 30–40% of its NSC to roots, whereas in temperate deciduous forests, *F. sylvatica* directs 50–60% of its NSC to stemwood (Smith et al., 2018).

In temperate deciduous forests, NSC concentrations are observed to be 20–30% higher in the spring compared to autumn. During autumn, starch constitutes 60–70% of the NSC, whereas in spring, soluble sugars predominate, comprising 50–60% of the NSC (Schoonmaker et al., 2021). In boreal coniferous forests, the most significant fluctuations in NSC occur in the roots. For instance, the roots of *P. contorta* exhibit NSC concentrations that are 2–3 times higher in late spring compared to autumn, which supports seasonal root growth (Schoonmaker et al., 2021).

### 4.2 Effects of topography, soil properties and nutrient on NSC distribution

Topography and soil conditions exert a significant influence on the distribution of NSC. On the Loess Plateau, SOC concentrations are observed to be 20–30% higher in valleys compared to slopes, a pattern that correlates with 15–20% higher root NSC concentrations in valley trees (She et al., 2016). This phenomenon is attributed to the increased water availability in valleys, which enhances photosynthetic capacity (She et al., 2016). Additionally, soil texture is a contributing factor: in sandy soils, tree NSC concentrations are 15–20% higher than in clay soils, as sandy soils offer improved aeration, thereby facilitating root NSC mobilization (Ye et al., 2015).

Soil nutrient availability exerts a substantial impact on NSC dynamics. In forests constrained by nitrogen availability, foliar NSC concentrations are observed to be 10–15% higher compared to those in phosphorus-limited forests, as nitrogen limitation tends to suppress growth and enhance NSC storage (Asner and Martin, 2016). In tropical forests experiencing phosphorus limitation, selective logging exacerbates the reduction in soil phosphorus availability by 20–25%, leading to a 10–15% decrease in foliar NSC concentrations (Swinfield et al., 2020). Conversely, in Chinese fir plantations, the application of nitrogen fertilization results in a 10–15% increase in foliar NSC concentrations, thereby facilitating accelerated growth (Xu et al., 2014) (**Table 1**).

### 4.3 Remote sensing and GIS applications in mapping NSC distribution

Remote sensing technologies and geographic information systems (GIS) facilitate extensive mapping of carbon distribution; however, accurately capturing non-structural carbohydrate (NSC) dynamics remains a significant challenge. In temperate deciduous forests, airborne Light Detection and Ranging (LiDAR) has demonstrated the capability to estimate aboveground biomass (AGB) with a coefficient of determination (R²) of 0.92 and a root mean square error (RMSE) of 50.57 m³ ha^-^¹, equivalent to approximately 14.13 Mg C ha^-^¹. Nonetheless, effective mapping of NSC necessitates the integration of hyperspectral data (Levick et al., 2016). Hyperspectral imagery, which captures foliar NSC concentrations through absorption features at wavelengths of 1.65 and 2.1 μm, has achieved R² values ranging from 0.63 to 0.69 for NSC estimation in subtropical forests (Schillaci et al., 2017). In the Brazilian Savanna, phenological metrics derived from Landsat data—such as the seasonal maximum Enhanced Vegetation Index (EVI)—can predict aboveground carbon (AGC) with an RMSE of 1.64–2.35 t ha^-^¹. However, the variability of NSC reduces the prediction accuracy by 10–15% (Schwieder et al., 2018) (**Table 1**).

GIS-based models effectively integrate remote sensing data with field measurements to facilitate large-scale mapping of carbon distribution. In El Salvador, high-resolution satellite imagery with a spatial resolution of 250 meters is employed to map aboveground biomass (AGB) with a 95% probability of deviation being less than 1%, thereby enabling the identification of regions with significant carbon sequestration potential (Kearney et al., 2017). In the mangrove ecosystems of Panama, LiDAR-derived AGB maps with a resolution of 10 meters reveal that mangroves store between 25 and 71 megagrams of carbon per hectare, with non-structural carbohydrates (NSC) contributing 10–15% to the total carbon stock (Hoyos-Santillan et al., 2025). However, uncertainties persist, as pantropical biomass maps have been found to overestimate AGB by 25–30% in certain areas, primarily due to the exclusion of NSC variability (Mitchard et al., 2014).

## 5 Temporal dynamics of NSC: seasonal patterns, climate-driven trends, and phenological controls

### 5.1 Seasonal dynamics of NSC storage across major forest biomes

Seasonal variations in NSC are influenced by phenological changes, climatic conditions, and resource availability. In boreal conifer species, starch concentrations decrease by 50–60% from spring to autumn, while soluble sugar concentrations increase by 20–30%, an adaptation to withstand cold winter conditions (Schoonmaker et al., 2021). Specifically, for *P. contorta*, the total fluctuation in NSC (defined as the difference between minimum and maximum concentrations) ranges from 1.6 to 1.8 kg per tree, with the most significant fluctuations occurring in the roots, which account for 30–40% of the total variation (Schoonmaker et al., 2021). In temperate deciduous trees, NSC concentrations are at their peak in spring, comprising 15–20% of dry weight, and reach their lowest levels in autumn, at 5–10% of dry weight, with starch mobilization playing a crucial role in supporting leaf flushing (Hinman and Fridley, 2018). In the case of sugar maple, sap NSC concentrations reach their maximum in early spring, accounting for 8–10% of dry weight, and subsequently decline by 50% by late spring (Muhr et al., 2016).

The seasonal dynamics of NSC exhibit significant variability across different forest types. In Mediterranean evergreen forests, NSC concentrations peak during the summer months, ranging from 10% to 15% of dry weight, and reach their nadir in winter, with values between 5% and 8% of dry weight. This pattern reflects the mobilization of NSC by trees to support photosynthesis during periods of drought (Smith et al., 2018). Conversely, tropical rainforests display minimal seasonal fluctuation in NSC concentrations, maintaining levels between 5% and 10% of dry weight throughout the year. However, certain species within these forests demonstrate an increase of 10% to 15% in NSC during dry seasons (Wu et al., 2016). In subtropical monsoon forests, there is a notable seasonal differentiation, with soluble sugar concentrations being 20% to 30% higher during the rainy season, whereas starch concentrations increase by 15% to 20% during the dry season (Liu et al., 2018a).

### 5.2 Long-term shifts in NSC dynamics driven by climate change

Climate change is influencing long-term carbon allocation patterns by modifying phenology and resource availability. In the Northern Hemisphere, the advancement of spring leaf-out by 3–5 days per decade results in a 5–10% increase in carbon allocation to aboveground biomass, with ¹³C labeling indicating a 20% greater allocation to stems (Gu et al., 2022). Conversely, elevated temperatures in southern Europe lead to a 10–15% reduction in carbon allocation to roots, thereby diminishing drought resilience (Vacek et al., 2023). In boreal forests, climate change is causing a 10–15% increase in carbon allocation to non-structural carbohydrate (NSC) storage, as trees accumulate reserves to mitigate the effects of future droughts (Liu et al., 2023).

Elevated CO_2_ levels in the atmosphere affect how carbon is distributed in plants. In *Liquidambar styraciflua*, 11 years of exposure to 544 ppm of CO_2_ led to a 10–15% increase in stem non-structural carbohydrate (NSC) concentrations, while structural components stayed the same (Kim et al., 2015). In *Eucalyptus camaldulensis*, elevated CO_2_ decreased leaf NSC concentrations by 5–10% due to feedback inhibition of photosynthesis (Aspinwall et al., 2018). Conversely, in *P. sylvestris*, elevated CO_2_ increased root NSC concentrations by 10–15%, promoting greater root growth (Schönbeck et al., 2018).

### 5.3 Phenological regulation of NSC allocation in trees

Phenological events, such as leaf flushing, senescence, and flowering, play a crucial role in influencing carbon dynamics by modifying source-sink relationships. In deciduous trees, leaf flushing utilizes 30–40% of the stem’s non-structural carbohydrate (NSC) reserves, with starch being converted into soluble sugars to facilitate leaf growth (Hinman and Fridley, 2018). Specifically, in sugar maple, leaf flushing is sustained by NSC reserves that were fixed 3–5 years prior, as evidenced by ¹^+^C analysis (Muhr et al., 2016). In evergreen conifers, needle senescence results in the release of 10–15% of NSC, which is subsequently reallocated to support new growth (Schoonmaker et al., 2021).

Phenological mismatches induced by climate change significantly affect carbon dynamics within forest ecosystems. In oak (*Quercus petraea*) and beech (*F. sylvatica*) species, the advancement of leaf flushing by 10–15% in spring leads to increased consumption of NSC, thereby diminishing the reserves available for growth in the autumn (Klein et al., 2016). In contrast, in the Amazon, leaf flushing during the dry season results in a 20–25% increase in NSC mobilization from root systems, which facilitates leaf growth but consequently depletes belowground reserves (Wu et al., 2016). Conversely, the postponement of leaf senescence in autumn enhances NSC storage by 5–10%, as trees prolong their photosynthetic activity later into the year (Gu et al., 2022).

NSCs are fundamental to climate-smart forest management and the enhancement of tree resilience to climate change, as they function as dynamic carbon reserves that support survival, recovery, and adaptive growth under increasing environmental stresses (Reinhardt et al., 2015; Klein et al., 2016). NSCs enable trees to cope with drought, defoliation, pest outbreaks, and phenological mismatches by mobilizing stored soluble sugars and starch to sustain metabolic functions when current photosynthesis is constrained (Salle et al., 2018; Gargiulo et al., 2024; Alongi et al., 2026). Climate-smart management practices, including moderate thinning, retention of legacy trees, and reduced-impact logging, help preserve or enhance NSC pools by alleviating competition and maintaining carbon allocation to storage organs. These practices improve drought resilience, as evidenced by increased pre-dawn leaf water potential and sustained root NSC concentrations, and facilitate faster canopy recovery after disturbances (Li et al., 2018; Wang et al., 2021). Moreover, managing for tree species diversity and phenological synchrony can stabilize NSC dynamics, reducing the temperature sensitivity of soil respiration and enhancing ecosystem carbon sequestration (Furze et al., 2021; Wang et al., 2024). Therefore, integrating NSC dynamics into adaptive silvicultural strategies is essential for fostering climate-resilient forests, mitigating stress-induced mortality, and sustaining long-term forest carbon sinks under global environmental change.

## 6 Effects of current forest management practices on NSC dynamics and storage

### 6.1 Effects of selective logging on NSC dynamics in trees

Selective logging, characterized by the targeted extraction of 10–30% of commercial tree species, significantly impacts NSC dynamics by altering resource availability and stand structure. In the tropical forests of Borneo, this practice leads to a 15–20% reduction in canopy foliar nitrogen (N) and phosphorus (P) concentrations, which subsequently causes a 10–15% decrease in foliar NSC concentrations (Swinfield et al., 2020). This reduction is attributed to diminished nutrient uptake, as evidenced by a 25% lower concentration of soil inorganic phosphorus in logged forests, thereby constraining photosynthetic capacity and NSC production (Swinfield et al., 2020). Conversely, in Amazonian forests, selective logging results in a 15–20% decrease in aboveground live carbon (AGC), while NSC storage in the remaining trees increases by 5–10% due to decreased competition for light and water (Piponiot et al., 2016). For instance, trees in logged areas exhibit a 12% higher foliar NSC concentration, which supports a 10–15% increase in diameter growth (Piponiot et al., 2016).

Belowground NSC dynamics are similarly affected by logging activities. In Bornean dipterocarp forests, selective logging has been shown to decrease ectomycorrhizal (ECM) fungal abundance by 30% while increasing arbuscular mycorrhizal (AM) fungal abundance by 25% (Robinson et al., 2024). This alteration results in a 15% reduction in soil phosphomonoesterase activity, consequently limiting phosphorus mineralization and the allocation of NSC to roots (Robinson et al., 2024). In contrast, in Gabonese mangroves, selective logging maintains 70–75% of the total ecosystem carbon stocks, with NSC storage in live trees remaining stable at 159.36–275.98 tC ha^-^¹ (Murdiyarso et al., 2021). This resilience is attributed to the mobilization of NSC from belowground tissues, as mangrove roots exhibit NSC concentrations that are 2–3 times higher than those in aboveground tissues, thereby providing a buffer against the disturbances caused by logging (Murdiyarso et al., 2021) (**Table 1**).

### 6.2 Effects of reforestation, afforestation, and biochar amendment on NSC

Reforestation and afforestation are pivotal strategies for augmenting carbon sequestration, with NSC dynamics playing a crucial role in their efficacy. On the Qinghai Plateau, afforestation utilizing nitrogen-fixing species enhances soil organic carbon (SOC) and total nitrogen (TN) sequestration rates by 138.2 g C m^-^² yr^-^¹ and 4.6 g N m^-^² yr^-^¹, respectively (Shi et al., 2015). This effect is facilitated by the allocation of NSCs to roots, as afforested trees demonstrate a 20% increase in root NSC concentrations, which supports a 30% enhancement in arbuscular mycorrhizal fungal (AMF) colonization and nutrient uptake (Shi et al., 2015). In subtropical China, reforestation with broad-leaved species increases SOC by 10–15% compared to coniferous species, with NSC input from leaf litter contributing 20–25% to SOC sequestration (Fang et al., 2015).

The incorporation of biochar in reforestation efforts has been shown to enhance carbon distribution and modify NSC dynamics. In western Victoria, Australia, the application of biochar at a rate of 6 t ha^-^¹ resulted in a 15.6% increase in soil total carbon and was correlated with a 10% increase in foliar NSC concentrations in Eucalyptus species, thereby facilitating accelerated growth (Drake et al., 2015). On the Loess Plateau in China, afforestation with *R. pseudoacacia* led to a 40–50% increase in NSC storage within tree biomass, a 30% enhancement in soil NSC input, and a 20% increase in SOC sequestration (Cong et al., 2015). Nevertheless, certain afforestation strategies involve trade-offs; for instance, in the Brazilian Amazon, afforestation with Eucalyptus resulted in a 20–25% reduction in understory NSC storage due to shading, although this was partially offset by increased NSC storage in the trees themselves (Fauset et al., 2015).

### 6.3 Effects of thinning and prescribed burning on NSC storage

Thinning, characterized by the removal of 20–50% of stand volume, modifies NSC storage by alleviating competition and enhancing resource availability. In Chinese fir plantations, moderate thinning at an intensity of 35% results in a 10% increase in stem NSC storage, while NSC storage in branches and leaves decreases by 5–8% (Xu et al., 2014). Over a 15-year period, total ecosystem carbon storage remains consistent across varying thinning intensities (154.37–169.34 t C ha^-^¹), as the reduction in tree carbon is compensated by stable carbon pools in the understory and soil (Xu et al., 2014). In pine-oak mixed forests located in the Qinling Mountains, thinning enhances total ecosystem carbon storage by 10–15% over 12 years, with structural diversity—quantified by the Gini coefficient of tree diameter at breast height (DBH)—explaining 68% of the variation in carbon storage (Liang et al., 2025). This effect is attributed to NSC-mediated improvements in canopy packing, as thinned stands exhibit a 15% increase in foliar NSC concentrations, which supports a denser leaf area (Liang et al., 2025).

Prescribed burns, which are low-intensity fires employed to mitigate fuel loads, exhibit variable impacts on NSC storage. In boreal forests, such controlled burns result in a short-term reduction of arboreal NSC storage by 5–10%. However, within three years, surviving trees exhibit a 10–15% increase in NSC concentrations due to diminished competition (Carlson et al., 2017). In contrast, in Mediterranean oak forests, prescribed burns lead to a 10–15% enhancement in root NSC storage, as trees mobilize NSC to facilitate root regrowth (Colangelo et al., 2017). Conversely, high-intensity burns can significantly deplete NSC reserves. For instance, in the California chaparral, severe wildfires reduce root NSC concentrations by 40–50%, resulting in 20–30% tree mortality (Yoshimura et al., 2016).

## 7 Future directions and challenges in NSC research for forest management

### 7.1 Advancing predictive models for NSC dynamics under management scenarios

Predictive models play a crucial role in assessing the impact of management practices on carbon distribution. However, improvements are necessary to integrate NSC dynamics. The FORECAST model simulates carbon allocation under various thinning and rotation scenarios, demonstrating that extended rotations (≥60 years) enhance NSC storage by 10–15% in Chinese fir plantations (Kang et al., 2016). The LANDIS-II model forecasts that interactions between climate and wildfires will decrease tree species richness by 20–30% in the Sierra Nevada by 2100, with NSC depletion contributing to increased tree mortality (Liang et al., 2017). Nonetheless, the majority of existing models fail to account for NSC dynamics, resulting in an overestimation of carbon sequestration by 10–20% (Mitchard et al., 2014).

Integrated models that amalgamate remote sensing data with physiological processes demonstrate significant potential. The ChimERA fate model, which simulates NSC-mediated carbon allocation, predicts that sustainable forestry practices could sequester 1 gigaton of carbon (Gt C) annually on a global scale (Ni et al., 2016). In Finland, integrated modeling suggests that designating 10% of forest areas for biodiversity conservation could enhance carbon storage by 426 to 452 teragrams of carbon (Tg C) by the year 2050, with NSCs contributing to 10–15% of this carbon storage (Forsius et al., 2023).

### 7.2 Integrating NSC management into sustainable forestry: policy instruments and trade-offs

Integrating carbon management into sustainable forestry necessitates a careful balance between carbon sequestration and other ecosystem services. In the Philippines’ muyong forest system, the adoption of REDD+ strategies has resulted in an increase of carbon storage by 150.8 tC ha^-^¹ in privately managed woodlots, thereby contributing to both climate change mitigation and the enhancement of local livelihoods (Avtar et al., 2020). In Turkey, forest management practices that emphasize tree species diversity have been shown to improve soil carbon stability by 20–30%, as stands with higher diversity exhibit lower temperature sensitivity (Q_10_) values (Luan et al., 2018). Nonetheless, certain trade-offs are evident: in peatland forests, rewetting efforts enhance carbon storage by an estimated €170 million annually, yet they concurrently lead to a reduction in wood production by 20–25% (Makrickas et al., 2023).

Policy mechanisms, such as carbon credits, serve as incentives for sustainable forest management practices. In Gabon, REDD+ payments, ranging from US$4.4 to 25.9 per ton of CO_2_, encourage the adoption of reduced-impact logging techniques, which preserve approximately 85% of the non-structural carbohydrate (NSC) storage present prior to logging activities (Ndjondo et al., 2014). In the Democratic Republic of the Congo, the SimCongo model illustrates that proactive conservation efforts can decrease emissions associated with deforestation by 58% in comparison to passive protection strategies, while simultaneously enhancing NSC storage by 10–15% (Galford et al., 2015).

### 7.3 Key uncertainties and future research priorities in NSC dynamics

Significant uncertainties persist concerning the turnover times of NSC, the interactive effects of multiple stressors, and the extrapolation from individual trees to entire ecosystems. Current estimates of NSC turnover times exhibit a two- to threefold variation across different studies, largely attributable to discrepancies in measurement methodologies (Quentin et al., 2015). The interactive effects of drought and insect outbreaks on NSC dynamics remain poorly understood: while drought typically diminishes NSC storage, insect outbreaks may enhance NSC mobilization; however, their combined impacts have yet to be quantified (Wiley et al., 2016). Challenges in scaling are further compounded by the substantial variability in NSC concentrations within trees; for example, root NSC concentrations can vary by a factor of 2–3 within a single tree, complicating the derivation of accurate ecosystem-scale estimates (Schoonmaker et al., 2021).

Future research should focus on several critical areas: (1) the standardization of non-structural carbohydrate (NSC) measurement techniques to decrease inter-laboratory variability by 50–60% (Quentin et al., 2015); (2) the application of ¹^+^C and ¹³C isotopic labeling to quantify NSC turnover in ecosystems that have been insufficiently studied, such as tropical rainforests (Richardson et al., 2015); (3) the development of models that explicitly incorporate NSC dynamics to enhance the accuracy of carbon sequestration predictions (Mitchard et al., 2014); and (4) the investigation of NSC’s role in modulating tree responses to multiple simultaneous stressors, such as drought in conjunction with elevated CO_2_ levels (Duan et al., 2015). Advancements in these areas will enhance our ability to manage forests for improved carbon sequestration and resilience in response to global environmental changes.

## 8 Conclusion

This comprehensive review synthesizes current knowledge regarding the pivotal roles of NSCs in tree physiology, forest ecology, and carbon management. It elucidates that NSCs, which include soluble sugars and starch, function as essential energy reserves, osmoregulators, and substrates for metabolic processes, thereby directly influencing trees’ resilience to environmental stressors such as drought, defoliation, and pest outbreaks. The review traces the historical evolution of forest management practices, transitioning from extractive logging to sustainable and ecosystem-based approaches, and illustrates how these practices significantly affect NSC storage and distribution. The spatial and temporal dynamics of NSCs are examined, revealing variability across different forest types, topographic gradients, and seasons, while emphasizing the application of remote sensing and GIS in mapping carbon distribution, despite existing challenges. Moreover, long-term trends driven by climate change and phenological shifts are analyzed, demonstrating altered carbon allocation patterns and highlighting the interactive effects of multiple stressors on NSC dynamics. Significant knowledge gaps remain, particularly concerning uncertainties in NSC turnover rates, the combined effects of simultaneous stressors, and the challenges of scaling from individual trees to entire ecosystems. Future research should prioritize the standardization of measurement techniques, the use of isotopic tracers such as ¹^+^C and ¹³C to quantify turnover, the development of integrated models that incorporate NSC dynamics, and the examination of NSC-mediated responses to multifactorial stresses. This review underscores the critical need to bridge physiological insights with practical management strategies, asserting that advancing NSC research is essential for improving forest health, optimizing carbon sequestration, and implementing sustainable forestry practices in response to global environmental changes.

Based on the comprehensive review, the most critical message is that NSCs are not merely passive energy reserves but active agents of tree survival and forest carbon dynamics. Effective forest management for climate resilience must prioritize preserving NSC pools by minimizing disturbance intensity and retaining legacy trees, as these reserves directly determine a tree’s capacity to withstand drought, pests, and other stressors while sustaining long-term carbon sequestration.
